# Supplementary material for: Timepoint-Specific Benchmarking of Deep Learning Models for Glioblastoma Follow-Up MRI
Source: Cancers (Basel). 2025 Dec 22;18(1):36. doi: 10.3390/cancers18010036 (PMC12784772; doi:10.3390/cancers18010036)
Supplement: Supplementary file 1 [file cancers-18-00036-s001.zip › cancers-4015131-supplementary.pdf]

Supplementary Document

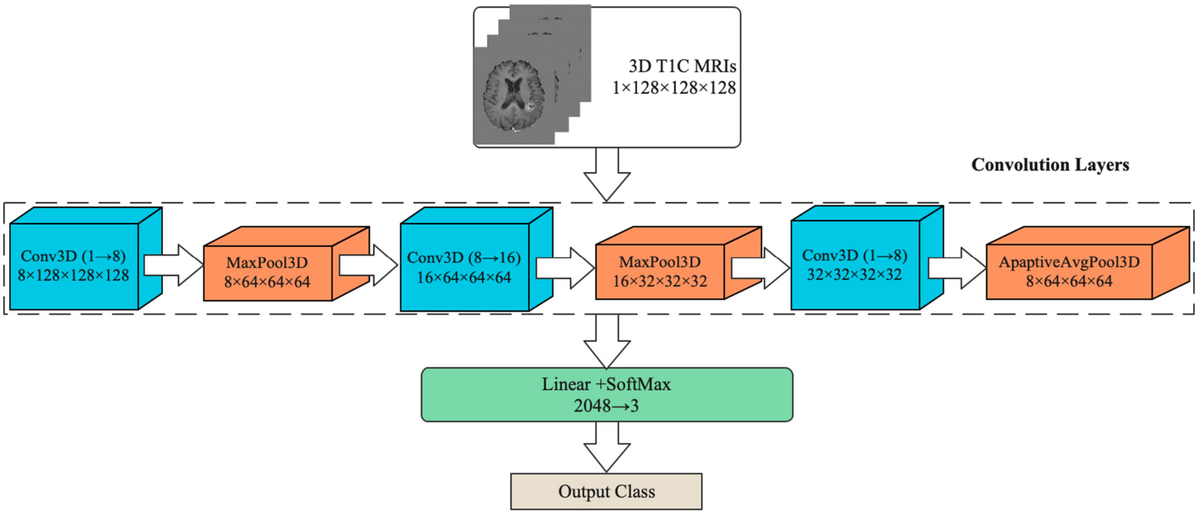

Figure S1. CNN architecture. A compact 3D-CNN was employed to classify preprocessed T1-contrast (T1C) MRI volumes (input 1×128×128×128). Sequential Conv3D–BatchNorm–ReLU blocks with increasing channels (8→16→32) made up the network. Translation-equivariant volumetric features were produced by convolutions using 3×3×3 kernels (stride 1, padded). MaxPool3D (2×2×2) improved efficiency and resilience to minor misalignments by expanding the receptive field and decreasing spatial resolution after the first two blocks. To produce three class probabilities, features were combined, flattened to a 2048-D vector, and then sent to a linear-softmax head. Through weight sharing and pooling, this design strikes a balance between maintaining the anatomical context and optimizing parameters.

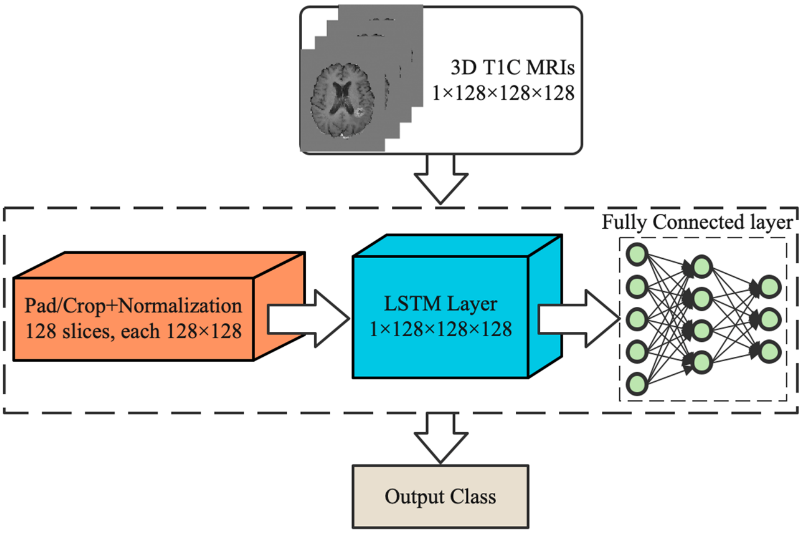

Figure S2. LSTM architecture. Each T1C volume was treated as an ordered sequence of 128 axial slices. The slices were padded/cropped and z-score normalized to 128×128. At time step t, the vectorized slice (or its low-level features) was fed to a unidirectional LSTM, which used its gating mechanism to decide what new information to store, what past information to discard, and what to expose to the next step. The sequence representation (final hidden state) was passed to a fully connected layer with SoftMax to produce class probabilities. This formulation preserves superior–inferior anatomical continuity while remaining parameter-efficient compared with full 3D convolutions.

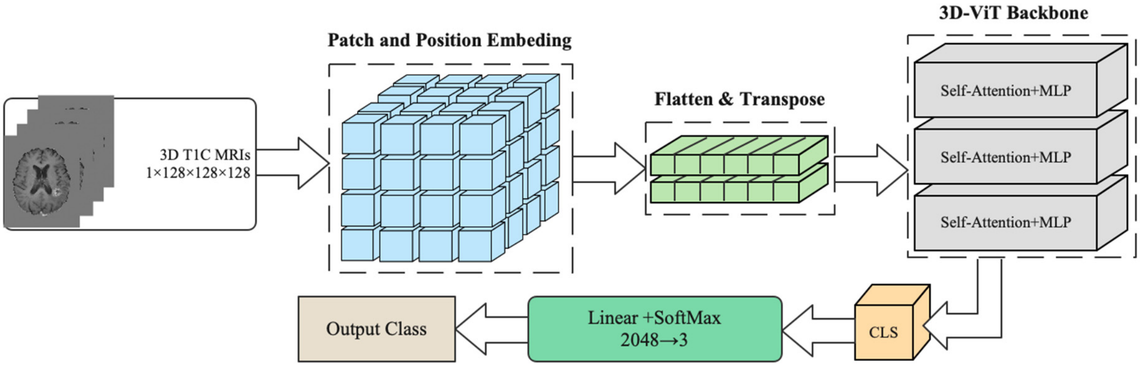

Figure S3. 3D-ViT architecture. Each preprocessed T1C volume ($1 \times 128 \times 128 \times 128$) was partitioned into non-overlapping 3D patches. Each patch was linearly projected to an embedding and augmented with a learnable positional embedding; a class (CLS) token was prepended. The resulting token sequence (after flattening/transposition) was processed by a stack of Transformer encoder blocks (multi-head self-attention + MLP with residual connections and layer normalization), allowing global, cross-patch interactions. The final CLS embedding was passed to a linear–SoftMax head (2048→3) to produce class probabilities. By operating on tokens rather than pixels/voxels, 3D-ViT captured long-range volumetric dependencies while remaining architecture-agnostic to spatial size.

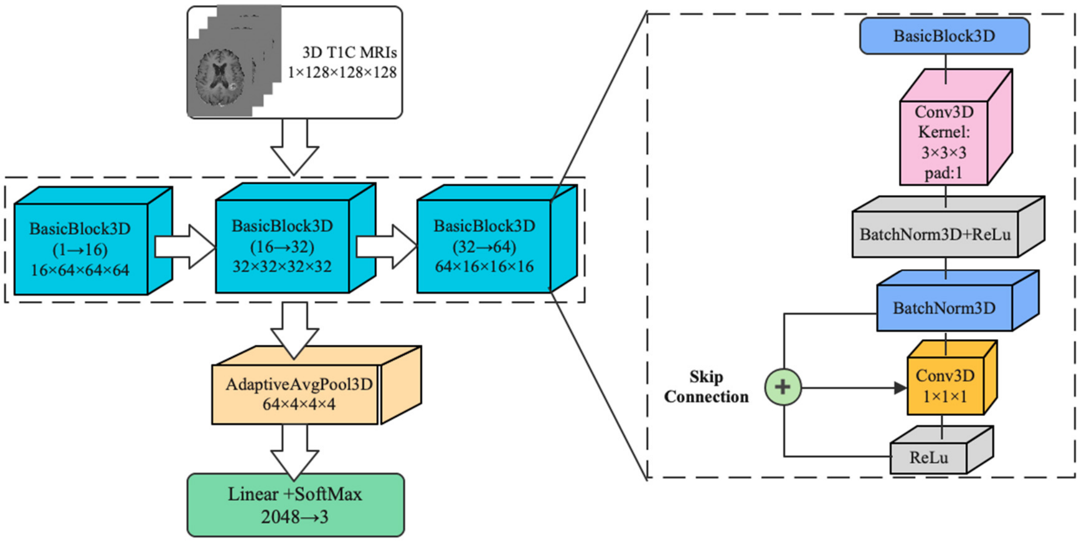

Figure S4. 3D-ResNet architecture. A volumetric 3D-ResNet is developed, which each BasicBlock3D contains Conv3D→BatchNorm→ReLU→Conv3D→BatchNorm, plus an identity skip; when channel or resolution changes, the skip uses a $1 \times 1 \times 1$projection with stride to align dimensions. The backbone comprised three stages with increasing channels (8→16, 16→32, 32→64); the first block of each stage downsampled spatially (stride 2), yielding feature maps of roughly $64^3 \rightarrow 32^3 \rightarrow 16^3$. Features were aggregated by AdaptiveAvgPool3D to a fixed grid and flattened to a 2048-dimensional vector, then passed to a linear–SoftMax head to produce three class probabilities. This residual design preserves volumetric context with modest parameter growth and mitigates vanishing-gradient effects compared with plain 3D CNNs.

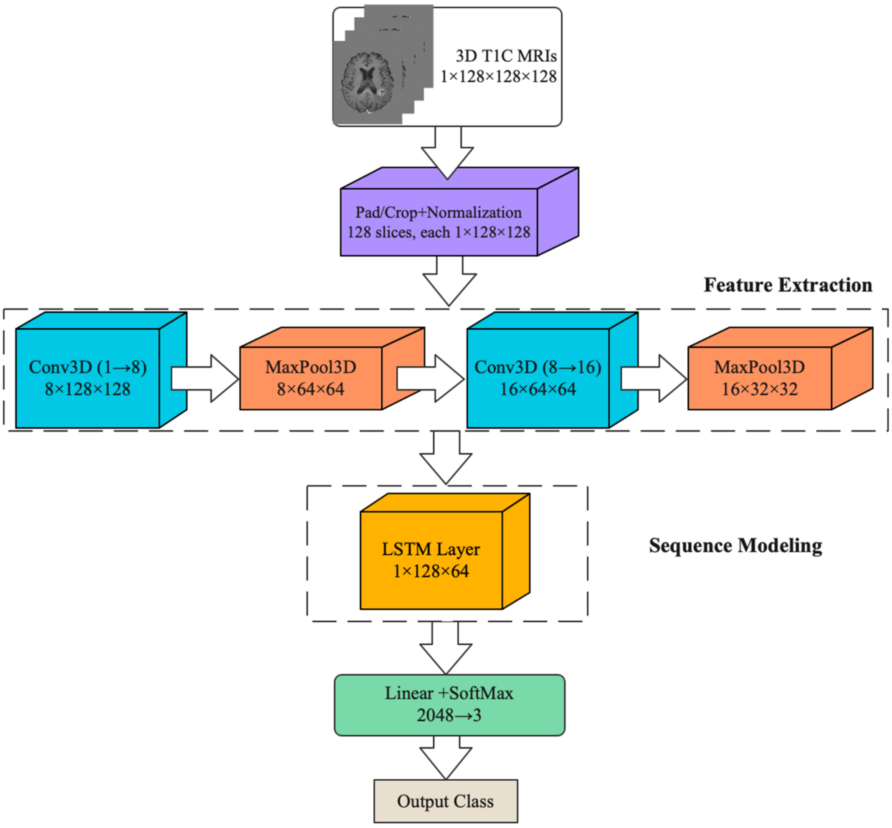

Figure S5. CNN+LSTM architecture. Convolutional encoders excel at extracting local spatial patterns, whereas recurrent networks, LSTM units—model ordered dependencies. For volumetric MRI, treating the axial stack as a sequence allows us to couple slice-level feature extraction with slice-to-slice context modeling. As Fig. 5 shows, each preprocessed T1C volume ($1 \times 128 \times 128 \times 128$) was first padded/cropped and z-score normalized per slice. A shallow 3D CNN encoder (Conv3D–BatchNorm–ReLU blocks with interleaved MaxPool3D that downsampled in-plane while preserving depth) produced feature maps that were spatially aggregated over x–y to yield a sequence of 128 slice embeddings. This sequence was passed to a single LSTM layer, whose input, forget, and output gates regulated what new information to store, what past information to discard, and what to expose at each step; the final hidden representation was fed to a fully connected SoftMax classifier to output class probabilities. his CNN+LSTM design, inspired by prior work (e.g., Jang et al., 2018), captures fine-grained local cues within slices and long-range superior–inferior dependencies across the stack, while remaining more parameter-efficient than deep 3D-only models.

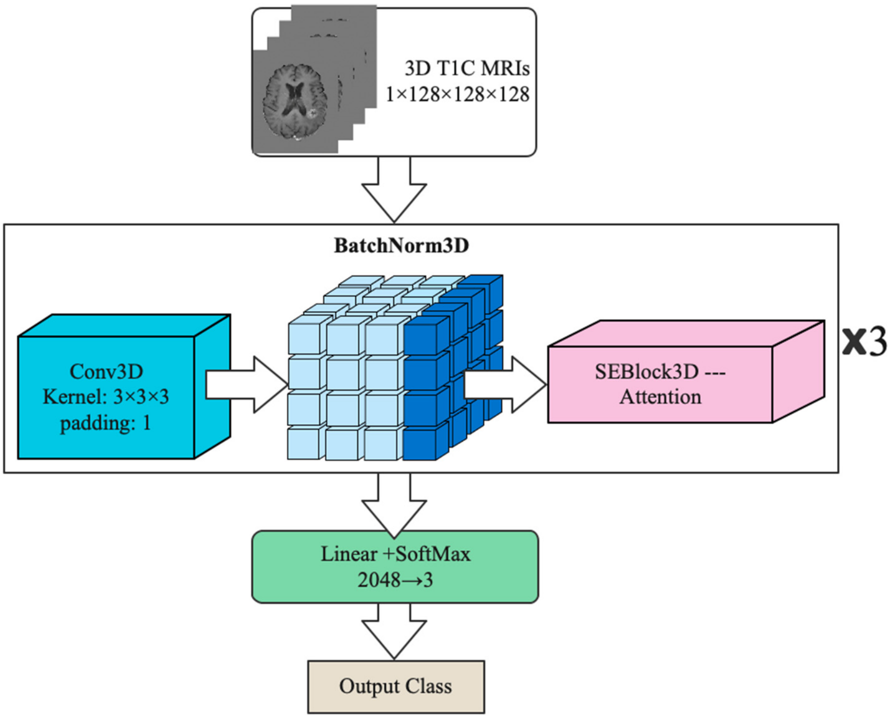

Figure S6. CNN+SE Attention architecture. Channel-attention improves convolutional features by letting the network emphasize informative channels and suppress distractors. Fig. 6 adopted a volumetric Squeeze-and-Excitation (SE) variant for T1C MRI: the model stacks three identical units, each consisting of a Conv3D layer (3 ×3 × 3, padding = 1) followed by BatchNorm3D and a 3D-SE block. The SE block first squeezes global spatial information via 3D global average pooling to form a channel descriptor, then excites channels through a small bottleneck (two fully connected layers with nonlinearity and a sigmoid gate) that re-weights the feature maps. After three such units, features are aggregated and flattened to 2048 dimensions, then passed to a linear–softmax head to produce three class probabilities. This architecture preserves local volumetric cues via convolutions while adaptively highlighting lesion-relevant channels through SE attention.

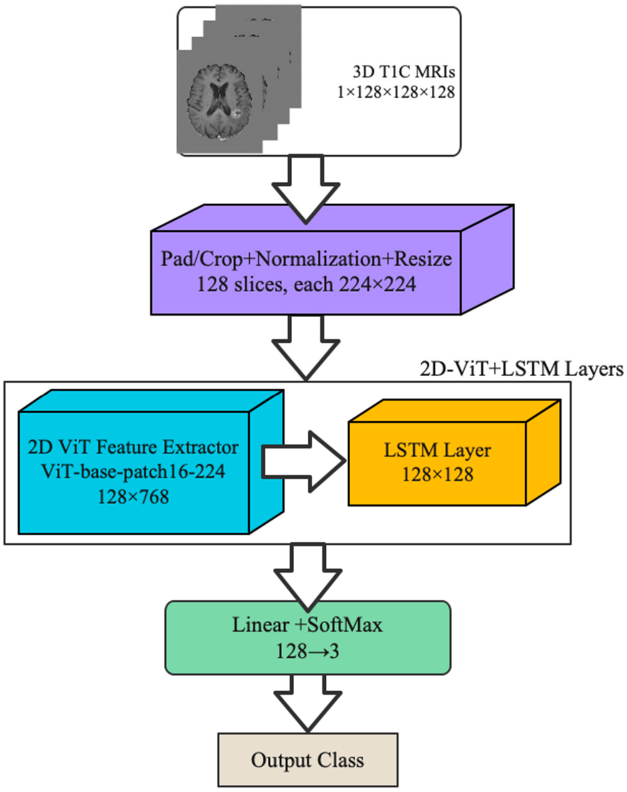

Figure S7. 2D ViT+LSTM. Transformers capture long-range dependencies by attending over patch tokens; a 2D Vision Transformer (ViT) applies this to single images, whereas volumes also require modeling relationships across slices**.** Fig. 7 shows that each preprocessed T1C volume (1 × 128 × 128 × 128) was treated as an ordered stack of 128 axial slices. Slices were pad/crop–normalized and resized to 224 × 224. A pretrained ViT-Base/16 model served as a per-slice feature extractor, yielding a 768-dimensional embedding (CLS token) for each slice. The resulting sequence (128 × 768) was fed to a unidirectional LSTM (hidden size = 128); the final hidden state was passed to a linear–SoftMax layer (128→3) to produce class probabilities. This design leverages ViT for global within-slice context and LSTM for slice-to-slice continuity, maintaining volumetric coherence with modest parameter cost.

Table S1. *Summary of Classification Performance (Accuracy, F1-score, and AUC) on First and Second Follow-up MRIs.*

	**First Follow-up**			**Second Follow-up**		
**Model**	**Accuracy**	**F1**	**AUC**	**Accuracy**	**F1**	**AUC**
**2DViT+LSTM (Batch=1)**	0.728 ± 0.0	0.2808 ± 0.0	0.4612 ± 0.0367	0.7024 ± 0.0001	0.2749 ± 0.0	0.4869 ± 0.0063
**2DViT+LSTM (Batch=8)**	0.6885 ± 0.0684	0.2676 ± 0.0228	0.5393 ± 0.028	0.7056 ± 0.0055	0.2865 ± 0.0201	0.5319 ± 0.0444
**3DViT (Batch=1)**	0.7181 ± 0.0154	0.3607 ± 0.0455	**0.5728 ± 0.0121**	0.724 ± 0.0244	0.4216 ± 0.0412	0.5879 ± 0.0541

**3DViT (Batch=8)**	0.7257 ± 0.0112	0.3688 ± 0.0452	0.5424 ± 0.0487	0.728 ± 0.0242	0.4162 ± 0.0445	0.6113 ± 0.0425
**CNN (Batch=1)**	0.6693 ± 0.0216	0.3329 ± 0.0209	0.4655 ± 0.0738	0.6324 ± 0.0645	0.3857 ± 0.0778	0.5537 ± 0.0322
**CNN (Batch=8)**	0.6818 ± 0.0292	0.3304 ± 0.0148	0.4817 ± 0.0378	0.6548 ± 0.0351	0.387 ± 0.0556	0.5813 ± 0.0154
**CNN+Attention (SE) (Batch=1)**	0.7061 ± 0.0213	0.3563 ± 0.0305	0.5439 ± 0.0473	0.6867 ± 0.0147	0.3878 ± 0.0251	0.5346 ± 0.0453
**CNN+Attention (SE) (Batch=8)**	0.7011 ± 0.0221	0.3183 ± 0.044	0.5378 ± 0.0299	0.7061 ± 0.0055	0.3886 ± 0.0065	0.5302 ± 0.0197
**CNN+LSTM (Batch=1)**	0.728 ± 0.0	0.2808 ± 0.0	0.5257 ± 0.0268	0.7057 ± 0.0058	0.2903 ± 0.0266	0.5412 ± 0.0199
**CNN+LSTM (Batch=8)**	0.728 ± 0.0	0.2808 ± 0.0	0.4704 ± 0.0614	0.7024 ± 0.0	0.2749 ± 0.0	0.4841 ± 0.0632
**Swin CNN (Batch=1)**	0.6341 ± 0.0864	0.3069 ± 0.0738	0.5251 ± 0.0376	0.6117 ± 0.084	0.2921 ± 0.0257	0.4754 ± 0.0118
**Swin CNN (Batch=8)**	0.728 ± 0.0	0.2808 ± 0.0	0.5 ± 0.0	0.7024 ± 0.0	0.2749 ± 0.0	0.5 ± 0.0
**LSTM (Batch=1)**	0.7305 ± 0.0043	0.2989 ± 0.0157	0.5008 ± 0.0723	0.7089 ± 0.0113	0.2649 ± 0.0174	0.4726 ± 0.0134
**LSTM (Batch=8)**	0.7255 ± 0.0043	0.2803 ± 0.0009	0.5161 ± 0.0511	0.7024 ± 0.0	0.2754 ± 0.0009	0.4638 ± 0.0655
**2D-Mamba (16 slices) (Batch=1)**	0.7024 ± 0.0	0.2749 ± 0.0	0.5556 ± 0.086	0.6358 ± 0.1154	0.2513 ± 0.0409	0.5336 ± 0.0893
**2D-Mamba (16 slices) (Batch=8)**	0.7329 ± 0.0084	0.2994 ± 0.0322	0.5604 ± 0.0375	0.7152 ± 0.0148	0.327 ± 0.0625	0.5496 ± 0.039
**2D-Mamba (50 slices) (Batch=1)**	0.6095 ± 0.1185	0.2413 ± 0.0395	0.5198 ± 0.0539	0.6706 ± 0.055	0.2642 ± 0.0185	0.4687 ± 0.0126
**2D-Mamba (50 slices) (Batch=6)**	0.735 ± 0.0005	0.3164 ± 0.0162	0.541 ± 0.025	0.7024 ± 0.0	0.2749 ± 0.0	0.5499 ± 0.0481
**2D-Mamba+CNN (Batch=1)**	**0.7451 ± 0.0261**	**0.4427 ± 0.1143**	0.5529 ± 0.0493	**0.7410 ± 0.025**	**0.5264 ± 0.0565**	0.6332 ± 0.0418
**2D-Mamba+CNN (Batch=2)**	0.716 ± 0.0349	0.3806 ± 0.0229	0.5416 ± 0.0676	0.7246 ± 0.011	0.4549 ± 0.0092	0.5647 ± 0.0431
**2D-Mamba+CNN (Batch=4)**	0.7278 ± 0.0297	0.3985 ± 0.0094	0.5464 ± 0.0342	0.7192 ± 0.011	0.4821 ± 0.0282	**0.6617 ± 0.0177**
**2D-Mamba+CNN (Batch=8)**	0.7183 ± 0.0152	0.3795 ± 0.019	0.5622 ± 0.0263	0.7055 ± 0.0279	0.4022 ± 0.0697	0.6075 ± 0.0233

**ResNet (Batch=1)**	0.6935 ± 0.0348	0.3331 ± 0.0852	0.4981 ± 0.0519	0.6705 ± 0.0305	0.38 ± 0.0459	0.5744 ± 0.015
**ResNet (Batch=8)**	0.7157 ± 0.0225	0.3605 ± 0.0401	0.5028 ± 0.0403	0.6708 ± 0.0312	0.4022 ± 0.0426	0.5448 ± 0.0156
**Swin Transformer (Batch=1)**	0.7009 ± 0.0535	0.2843 ± 0.0198	0.4806 ± 0.039	0.7056 ± 0.011	0.3065 ± 0.0033	0.4921 ± 0.0192
**Swin Transformer (Batch=6)**	0.3842 ± 0.2114	0.2037 ± 0.0657	0.4789 ± 0.0664	0.4071 ± 0.1748	0.1988 ± 0.062	0.4933 ± 0.0412

Table S2. *Summary of classification performance (Accuracy, F1-score, and AUC) on second follow-up MRI using a consistent batch size.*

	**First Follow-up**				**Second Follow-up**			
**Model**	**FLOPs**	**Params (M)**	**Batch Time (s)**	**Run Time (mins)**	**FLOPs**	**Params (M)**	**Batch Time (s)**	**Run Time (mins)**
**2DViT+LSTM (Batch=1)**	468.6238	5.6421	0.373 ± 0.096	2142.3667 ± 515.5379	468.6238	5.6361	0.3045 ± 0.0641	1766.9167 ± 544.7241
**2DViT+LSTM (Batch=8)**	468.6238	5.6421	3.364 ± 3.9684	2366.75 ± 1640.4337	468.6238	5.6361	2.5917 ± 2.7938	1905.4367 ± 1339.13
**3DViT (Batch=1)**	277.2708	88.1664	0.1753 ± 0.107	887.0333 ± 300.3485	277.2708	88.1664	0.1922 ± 0.0301	1170.7833 ± 343.5418
**3DViT (Batch=8)**	277.2708	88.1664	1.1301 ± 0.1592	948.2333 ± 143.8502	277.2708	88.1664	1.0392 ± 0.2969	1005.91 ± 206.0372
**CNN (Batch=1)**	12.3889	0.0832	0.044 ± 0.0107	529.7833 ± 80.0649	12.3889	0.0832	0.021 ± 0.0169	468.1633 ± 72.6496
**CNN (Batch=8)**	12.3889	0.0832	0.1439 ± 0.0943	407.6067 ± 100.5402	12.3889	0.0832	0.1304 ± 0.0934	390.8433 ± 51.1764
**CNN+Attention (SE) (Batch=1)**	44.821	2.4304	0.0662 ± 0.0332	596.93 ± 64.7776	44.821	2.4304	0.0382 ± 0.0141	454.77 ± 32.3068
**CNN+Attention (SE) (Batch=8)**	44.821	2.4304	0.1636 ± 0.0517	503.3133 ± 178.9891	44.821	2.4304	3.0258 ± 2.4875	1181.1167 ± 625.8477
**CNN+LSTM (Batch=1)**	5.6613	1.5707	0.0221 ± 0.0133	549.25 ± 7.9764	5.6613	1.2707	0.0192 ± 0.0125	430.42 ± 48.5683
**CNN+LSTM (Batch=8)**	5.6613	1.5707	0.1295 ± 0.0511	490.8967 ± 35.8705	5.6613	1.2707	0.0801 ± 0.0161	339.8367 ± 76.1156
**CNN+ShiftWindowPatch (Batch=1)**	**0.0255**	**0.0701**	0.0015 ± 0.0007	1114.4133 ± 187.3267	0.0255	0.0701	0.0022 ± 0.0009	1024.7733 ± 167.7931
**CNN+ShiftWindowPatch (Batch=8)**	**0.0255**	**0.0701**	**0.004 ± 0.0036**	**254.0 ± 114.0537**	**0.0255**	**0.0701**	**0.0023 ± 0.0007**	**233.3367 ± 101.256**

**LSTM (Batch=1)**	7.4189	20.3811	0.0039 ± 0.0013	483.2667 ± 47.298	7.4189	20.3811	0.0033 ± 0.0019	411.6133 ± 60.9606
**LSTM (Batch=8)**	7.4189	20.3811	0.0253 ± 0.0294	416.0533 ± 93.5496	7.4189	20.3811	0.0237 ± 0.0323	352.1033 ± 81.3641
**2D-Mamba (16 slices) (Batch=1)**	142.9558	24.5151	0.0808 ± 0.009	952.81 ± 248.1051	142.9558	24.5151	0.1279 ± 0.0879	1285.73 ± 666.0025
**2D-Mamba (16 slices) (Batch=8)**	142.9558	24.5151	0.197 ± 0.026	471.3967 ± 77.5497	142.9558	24.5151	0.1917 ± 0.0189	416.2267 ± 56.6206
**2D-Mamba (50 slices) (Batch=1)**	446.7358	24.5151	0.1544 ± 0.1406	2567.8533 ± 242.1994	446.7358	24.3734	0.0846 ± 0.0123	671.695 ± 30.1581
**2D-Mamba (50 slices) (Batch=6)**	446.7358	24.5151	0.2909 ± 0.0029	802.0667 ± 13.4718	446.7358	24.4206	0.2548 ± 0.0518	770.5533 ± 99.2479
**2D-Mamba+CNN (Batch=1)**	0.7776	24.2316	0.0801 ± 0.0864	787.97 ± 473.7224	0.7776	24.2316	0.0317 ± 0.0169	395.4667 ± 68.7862
**2D-Mamba+CNN (Batch=2)**	0.7776	24.2316	0.0349 ± 0.0076	631.7 ± 446.109	0.7776	24.2316	0.015 ± 0.003	326.1533 ± 88.3388
**2D-Mamba+CNN (Batch=4)**	0.7776	24.2316	0.0789 ± 0.0777	419.1267 ± 92.8542	0.7776	24.2316	0.1213 ± 0.1818	427.9933 ± 153.0907
**2D-Mamba+CNN (Batch=8)**	0.7776	24.2316	0.1595 ± 0.238	372.4667 ± 109.4993	0.7776	24.2316	0.1536 ± 0.2308	330.58 ± 90.0112
**ResNet (Batch=1)**	28.4955	0.6528	0.0784 ± 0.1079	511.38 ± 44.35	28.4955	0.7428	0.0169 ± 0.0027	416.0133 ± 43.1828
**ResNet (Batch=8)**	28.4955	0.6528	0.1052 ± 0.0244	331.75 ± 21.3322	28.4955	0.7428	0.1382 ± 0.0465	896.3633 ± 829.5617
**Swin Transformer (Batch=1)**	236.9559	7.8644	0.7694 ± 0.1436	1324.7833 ± 194.3059	236.9559	7.8644	0.9166 ± 0.3571	1404.7267 ± 300.9926
**Swin Transformer (Batch=6)**	236.9559	7.8644	0.6069 ± 0.0018	302.81 ± 8.3104	236.9559	7.8644	8.1024 ± 12.9558	265.25 ± 9.3466
